# Interpretable Skin Cancer Identification Using a Hybrid Deep Learning and XAI Framework on HAM10000

**DOI:** 10.3390/bioengineering13060677

**Published:** 2026-06-11

**Authors:** Bhagyashri S. Sonune, R. Udaya Kumar, K. Sankar, Puja S. Agrawal, Shon G. Nemane, Dhiraj P. Tulaskar, Manish Bhaiyya, Madhusudan B. Kulkarni

**Affiliations:** 1 Department of Computer Science, Kalinga University, Raipur 492101, Chhattisgarh, India; 2 Department of Artificial Intelligence and Data Science, Vel Tech High Tech Dr.Rangarajan Dr.Sakunthala Engineering College, Avadi, Chennai 600062, Tamil Nadu, India; 3 Department of Electronics and Communication Engineering, School of Electrical and Electronics Engineering, Ramdeobaba University, Nagpur 440013, Maharashtra, India; 4 Department of Electronics and Telecommunication Engineering, Shri Sant Gajanan Maharaj College of Engineering, Shegaon 444203, Maharashtra, India; 5 Manipal Institute of Technology, Manipal Academy of Higher Education (MAHE), Manipal 576104, Karnataka, India

**Keywords:** cancer detection, skin lesion classification, explainable artificial intelligence (XAI), image analysis, convolutional neural networks (CNNs), machine learning (ML), deep learning (DL)

## Abstract

Deep learning-based automated classification of dermatoscopic skin lesions has exhibited promising potential in diagnostics. However, two prominent issues need to be addressed before achieving high-quality diagnostic tools: inconsistent performance in the case of imbalanced classes and poor clinical interpretability of models. Even though some studies have attempted to leverage both deep and shallow learning by combining pretrained convolutional neural networks (CNNs)-based feature extraction with classical machine learning (ML) models, very few of them systematically explore several model combinations based on various clinically important metrics, such as F1-score, precision, recall, accuracy, etc., and utilize decision threshold calibration techniques. In this research, we present an evaluation of a systematic framework with threshold calibration for the comparison of several hybrid models on seven-class skin lesion classification (multi-class) on the HAM10000 dataset. In particular, we used deep features extracted from three pretrained CNN architectures, i.e., DenseNet201, InceptionV3 and EfficientNet-B4. These deep features were used as inputs for six different classical classifiers. As a result, we obtained 18 comparable hybrid models that were then systematically compared by multiple clinically relevant metrics: accuracy, macro-precision, macro-recall, macro-F1, ROC-AUC, Precision-Recall-AUC, and log loss. Also, fold-wise optimization of decision thresholds was performed, which was based on the maximization of the macro-F1 score. Finally, we found out that DenseNet201 with an SVM-RBF classifier yielded the highest performance among all 18 tested models, showing 90.88% accuracy, 90.7% macro-precision, and 0.921 ROC-AUC. To analyze the clinical plausibility, top-performing models were further explained with explainable artificial intelligence (XAI) techniques: Grad-CAM, LIME and Occlusion Sensitivity. Results show that the most successful models concentrated mostly on lesion-specific areas. Overall, this study contributes a reproducible hybrid-XAI model-selection framework rather than a single black-box classifier, supporting more transparent and clinically meaningful skin lesion diagnosis.

## 1. Introduction

The incidence of skin cancer is the highest of all cancers, and with an ongoing population of aged individuals who experience continual problems through their cumulative exposure to ultraviolet radiation and an increase in ‘suspicious’ lesion screening, the clinical burden has increased. Despite melanoma accounting for a smaller number of cancers when compared to non-melanoma cancers, it contributes to a much higher level of mortality, and by the time a patient presents with an advanced stage of melanoma, his/her prognosis has decreased significantly [1,2]. Dermoscopy provides an enhanced assessment of lesions, as dermoscopy demonstrates both the subsurface structure and pattern of the lesions. However, dermoscopy is only as accurate as the experience of the physician and may have inter-observer variation between medical professionals, and it is still difficult for the physician to visually differentiate between cutaneous melanoma skin cancers and cutaneous basal or squamous cell carcinoma skin cancers [3,4].

Deep learning, particularly the use of deep convolutional neural networks (CNNs), has garnered significant attention as a viable path to supporting dermoscopic image analysis. The advent of publicly available datasets has facilitated the advancement of this area; specifically, the HAM10000 dataset (10,015 dermoscopic images classified into seven of the most common categories for dermatological diagnosis) has provided a framework by which methods may be directly compared using reproducible procedures. End-to-end transfer learning uses a single pretrained backbone that has typically been fine-tuned to generate highly competitive results [5,6,7]. For instance, EfficientNet has been used extensively for applying transfer learning techniques for the multi-class recognition of lesions on the HAM10000 dataset [8,9,10]. In addition, studies have leveraged transformer-based architectures for vision tasks in dermoscopy by utilizing self-attention mechanisms to generate a global context that provides information about long-distance relationships between pixels within a dermoscopic image [11,12,13]. In addition, several other studies have combined architectural innovations, including using convolutional network modules to extract features with transformer networks, and developed loss functions that account for imbalances across classification categories, such as focal loss. These two forms of approaches have been explored for their potential to improve minority class recognition [14].

The translational value of many published systems suffers from three major limitations despite considerable advancements. First, most studies evaluate only a limited set of components (one backbone, one optimizer, and one training recipe), thus making it challenging to distinguish improvements due to architecture, data preprocessing, augmentation, or loss function design [15]. Second, because most clinical datasets (e.g., HAM10000) are clinically imbalanced with class overlap in some categories, the overall accuracy metric may mask clinical failure modes for less representative classes [16]. Third, “black box” predictions continue to prevent widespread adoption by patients and providers. Clinicians require a transparent rationale (or at least reliable visual evidence) before trusting or acting on outputs from an ML algorithm within a standard diagnostic workflow. This has led to a greater emphasis on the use of explainable artificial intelligence (XAI) techniques, such as gradient-weighted class activation maps (Grad-CAMs), local interpretable model agnostic explanations (LIMEs), and Shapley additive explanations (SHAPs) to show which areas of the image contributed to the algorithm’s decision-making process [17,18,19,20].

Deploying hybrid frameworks offers an immediate and practical means to address many of these challenges. A hybrid pipeline uses a pre-trained convolutional neural network (CNN) as a feature extractor and classifies using conventional machine learning (ML) classifiers on the embeddings produced by the CNN. Multiple classifiers can make it easier to establish accurate decision boundaries; examples exist of studies combining CNNs and support vector machine classifiers (SVMs) for dermatoscopic classification. Yet most hybrid studies only test a small number of model configurations (for example, few backbones and classifiers), and do not evaluate interpretability using multiple complementary XAI perspectives [21,22]. In response to the research gaps identified in this field, we present an evaluation of a set of different hybrid classifiers for interpretable classification of skin lesions into seven classes based on the HAM10000 dataset. Our focus is to evaluate 18 hybrid models consisting of three pre-trained CNNs (DenseNet201, InceptionV3, EfficientNet-B4) and six classical machine learning classifiers (SVM with a radial basis function kernel, XGBoost, Random Forest, KNN, AdaBoost, and Naive Bayes). The interpretability of these models is evaluated using Grad-CAM, LIME, and SHAP methods. Examples comparing our methods with other recent (2020) work on this topic can be found in Table 1, which also illustrates the wide range of options for constructing interpretable hybrid pipelines in this field.

In recent years, studies have provided support for the applicability of deep learning and hybrid approaches to classify medical images. In dermoscopy, both CNN and hybrid CNN-SVM methods have demonstrated competitive results for classifying lesions in the skin, whereas the fusion of features or multimodel methods has been attempted for multi-classification tasks. Such studies imply that the deep learning CNN features are capable of generating effective representations of lesions, and that classic ML algorithms or ensemble approaches can be effective, as long as these features are used as inputs. In other fields of clinical image data, the efficacy of similar deep convolutional neural network-based classification models has also been suggested in detecting abnormalities in endoscopic gastrointestinal images. However, most prior research work has evaluated a few model configurations or lacks a systematic comparison using sensitivity-oriented metrics, as well as an XAI analysis. Hence, this study aims to contribute to these efforts through a systematic evaluation of 18 hybrid CNN-ML pipelines using macro-metrics, optimal thresholds, and XAI visualization [9,21,23,24,25].

**Table 1:** Illustrates the wide range of options for constructing interpretable hybrid pipelines in this field.

Study	Dataset/Classes	Method	Validation/Split	Reported Performance	XAI Used	Direct Limitation Compared with Our Study
Ali et al., 2022 [26]	HAM10000/7 classes	EfficientNet B0–B7 transfer learning	Train/test split	Best EfficientNet-B4: 87.91% accuracy; F1 ≈ 87%	Not emphasized	Strong CNN benchmark, but no hybrid classifier comparison and limited XAI discussion
Tajerian et al., 2023 [27]	HAM10000/7 classes	EfficientNet-B1 web diagnostic model	Train/test split	84.3% accuracy; class F1 varied from 0.54 to 0.93	Not emphasized	Application-focused; limited model benchmarking and no multi-XAI validation
Xin et al., 2022 [28]	HAM10000 + clinical dataset	SkinTrans transformer	Dataset-level evaluation	94.3% accuracy on HAM10000; 94.1% on clinical dataset	Not emphasized	High accuracy transformer model, but not a hybrid CNN-ML/XAI framework
Keerthana et al., 2023 [23]	ISBI 2016/binary melanoma classification	Hybrid CNN + SVM	Public dataset evaluation	88.02% and 87.43% accuracy	Not emphasized	Binary task and different dataset; not directly comparable to 7-class HAM10000
Shah et al., 2024 [29]	ISIC 2018 and HAM10000	CNN + PSO + ML classifiers	Dataset-level evaluation	98.5% on ISIC 2018; 86.1% on HAM10000	XAI emphasized	Optimization-based pipeline; limited multi-backbone classifier benchmarking
Munjal et al., 2024 [30]	HAM10000/7 classes	SkinSage XAI using deep learning	Customized HAM10000 evaluation	Reported high diagnostic performance	Grad-CAM + LIME	XAI-focused, but mainly single-pipeline model rather than systematic 18-model comparison
Proposed study	HAM10000/7 classes	DenseNet201, InceptionV3, EfficientNet-B4 features + 6 ML classifiers; 18 hybrid pipelines	Stratified 5-fold cross-validation + macro-F1 threshold optimization	Best: DenseNet201 + SVM-RBF; 90.88% accuracy, 0.921 ROC-AUC, PR-AUC > 0.83, macro-F1 ≈ 0.86	Grad-CAM + LIME + Occlusion Sensitivity	Systematic hybrid-XAI benchmarking with multi-metric, imbalance-sensitive evaluation

## 2. Dataset and Pre-Processing

**Figure 1:**
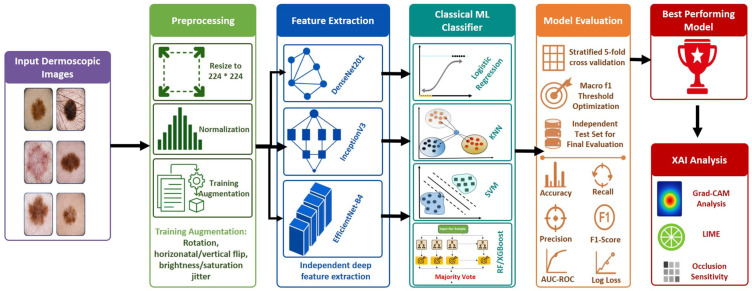
Proposed hybrid CNN–ML framework for dermoscopic skin lesion classification, showing preprocessing, deep feature extraction, classical ML classification, model evaluation, best-model selection, and XAI-based interpretation.

Due to its widespread popularity as a public dermoscopy database to evaluate automated skin lesion classification systems and the number of dermoscopy images available, the HAM10000 dataset was chosen as the primary benchmark for this study. The HAM10000 dataset consists of more than 10,000 dermoscopic images taken from clinical populations at the Medical University of Vienna and at the Skin Cancer Institute in Queensland, Australia. The HAM10000 dataset has been labeled/annotated into seven different diagnostic categories: Melanocytic Nevus (nv), Melanoma (mel), Basal Cell Carcinoma (bcc), Actinic Keratosis/Bowen’s Disease (akiec), Benign Keratosis-Like Lesions (bkl), Dermatofibroma (df), and Vascular Lesion (vasc). The main feature of HAM10000 is a significant amount of class imbalance (as shown in Figure 1). Specifically, the nv category represents the majority of samples, and minority categories such as df and vasc represent only a small percentage of the total datasets [7,21]. Although the significant class imbalance mirrors clinical prevalence, the overfitting of models to dominant categories may overestimate accuracy. Therefore, to ensure that the model’s performance is stable and comparable, the data will be stratified, and a macro-averaged evaluation method will be employed (using precision/recall and F1 score) for all lesion categories.

A standardized preprocessing workflow is applied to every dermoscopic image as part of an effort to establish consistency among all hybrid pipelines. The first step in this workflow is to resize each image to 224 x 224 pixels (to establish uniform spatial dimensions), while also preserving lesion structure and boundary information. This enables the extraction of features using pretrained convolutional neural network (CNN) encoders, such as DenseNet201, InceptionV3, and EfficientNet-B4 [31,32,33]. The second step in the preprocessing workflow involves normalization of each image using the mean and standard deviation values calculated from the ImageNet dataset. This creates consistency between the input intensity distribution of each image and the pretrained weights, improving the stability of the embedding and decreasing the sensitivity of the embedding to sudden changes in illumination. Random rotations (±20°), horizontal and vertical flips, and brightness and saturation jittering to simulate realistic variability experienced by dermoscopically acquired images are examples of optional augmentation that can be applied during training to provide a means to improve generalization and decrease overfitting [34]. Notably, artefact removal processes (e.g., hair removal, glare correction, or ruler-mark suppression) are not performed explicitly; therefore, although this process allows for reproducibility and does not introduce artefact-preprocessing bias, it may also create an impediment to the accurate representation of lesion morphology due to artefacts potentially obscuring lesion morphology and impacting extracted features, as addressed in the failure-mode analysis as a limitation [35].

## 3. Methodology

A hybrid deep learning framework has been developed based on a combination of classical ML classification techniques and frozen deep CNN feature extraction (see Figure 1). To combine the computational efficiency, robustness to medium-sized datasets, and enhanced interpretability of deep CNNs with classical ML classifiers, we propose using pre-trained CNN features as inputs to classical ML classifiers. In addition to improving interpretability through explainable AI, our hybrid method also has the potential to increase the generalisability of the model beyond what was previously achieved by using any one of these techniques alone. The approach has three stages: (i) diverse CNN architectures to extract deep features, (ii) a classically trained model using Deep CNN features, and (iii) explainable AI methods for model interpretability [36].

### 3.1. Convolutional Neural Network Feature Extractors

This research utilizes a combination of methods, in which already trained CNNs serve as a set of features rather than learning a feature set from an image. Three different types of backbone CNNs were selected (DenseNet201, InceptionV3, EfficientNet-B4) to enable multiple ways to view and analyze images taken using different methods (dermoscopy). These methods allow the use of the same set of pretrained (ImageNet) CNNs to aggregate multiple representations of images over the course of many different methods to create an aggregated representation that contains unique information not present in any single representation alone.

The selection of the three CNN backbones was done based on a complementary approach in terms of their philosophy in convolution operations, without making an attempt at proving their supremacy over current models. DenseNet201 is one such backbone because of its dense connection architecture, which enables the reuse of features across all layers, contributing towards maintaining small features, such as textures, borders, and pigmentations relevant to dermoscopy image analysis. InceptionV3 is another model whose architecture allows the capturing of both small- and large-scale features as a result of its multi-scale convolution modules, which is helpful when there are considerable variations in the sizes and morphologies of lesions within images. On the other hand, EfficientNet-B4 utilizes a scaling technique that balances model capacity, image resolution, and efficiency.

The classification layer in each CNN was removed, and the features were extracted from the final convolutional layer and transformed into a fixed-size vector through a process called Global Average Pooling (GAP). DenseNet201 was chosen for the high level of connectivity in the DenseNet model, as well as feature reuse, allowing for the identification of very small textures and patterns associated with the evaluation of lesions. The InceptionV3 model was chosen for its use of multiple convolutional kernels with layers of varying sizes, enabling it to take advantage of both large and small image structures in the same layer. The EfficientNet-B4 model was chosen due to the compound scaling methods used to balance model size with performance. The final size of the embedding generated by each CNN was 1920 for DenseNet201, 2048 for InceptionV3, and 1792 for EfficientNet-B4, and each embedding served as an input to the downstream classifiers, establishing the core of the combined hybrid evaluation framework.

### 3.2. Classical Machine Learning Classifiers

Once the deep features have been extracted from the images, the extracted embedding vectors will then be fed into an ensemble of classical ML classifiers for the completion of the final seven classes of lesion classification jobs. This hybrid design effectively decouples the representation learner (a pretrained convolutional CNN) from the classifier decision-making recommendation system (classifier), and therefore, it allows for efficient training (with stable optimization performance) while working on relatively medium-sized datasets, and facilitates systematic comparisons of the classifiers’ recommended decision boundaries in the same feature space. As part of being able to cover the full range of all different types of classification learning, we decided to incorporate the use of six very popular classifiers with a combination of linear, distances-based, ensemble and kernel-based classifiers.

The selected classical ML classifiers are used to incorporate different forms of decision boundaries into the embedding space provided by the CNN. In particular, KNN is considered a distance classifier; on the other hand, logistic regression and Linear SVM form linear classifiers, while SVM with the RBF kernel acts as a non-linear, kernel classifier. In addition, Random Forests and XGBoost can be considered bagging and boosting ensemble classifiers, respectively. Thus, the selected set of classifiers allows for an analysis of whether the obtained deep features can be better divided using linear, distance, tree-based, or kernel decision boundaries.

Around the world, KNN, as a base classifying approach, provides a clear example of how these models assign labels to an object according to nearby neighbors in the embedding space using the majority label of the neighbors. KNN does face challenges when working with high-dimensional representations because of its high sensitivity to distance scaling. Logistic regression offers a probabilistic approach based on linearity, which can be used for decision boundaries. As with KNN for high-dimensional models, logistic regression is also susceptible to underfitting when the separating characteristic of the lesion does not represent linearity.

The Random Forest classifier is an ensemble classifier of decision trees created through the use of bagging. While Random Forests may be well-suited to moderate amounts of non-linearity in the embedding space, the highest-dimensional embeddings often require much attention during hyper-parameter tuning. Support vector classifiers have been evaluated in both linear and SVM-RBF forms. SVM-RBF provides greater flexibility in defining non-linear decision boundaries and lends itself well to embedding spaces based on CNN. XGBoost is an extremely high-performance boosting classifier and excels with structured embedded data inputs due to the inherent regularization capabilities that allow it to capture deeply complex interactions between features. When taken together, these classifiers represent a wide-ranging, comparable set of models used to investigate the various combinations of classifiers that utilize deep embeddings for effective classification of skin lesions. All classifiers were trained using the same CNN-derived feature embeddings, and the same stratified cross-validation protocol to ensure a fair comparison among hybrid pipelines (see Table 2). The random state was fixed wherever applicable to improve reproducibility.

### 3.3. Training Protocol and Data-Splitting Strategy

In order to provide a bias-free assessment of our models without leaks, we decided to divide HAM10000 into a training subset and an independent test subset prior to the model training. In this work, we used the training subset, which consisted of 7902 images, while the independent test subset had 2113 images. The training subset comprised nv = 5364, mel = 834, bkl = 879, bcc = 359, akiec = 261, vasc = 113, and df = 92 images. The test set comprised nv = 1341, mel = 279, bkl = 220, bcc = 155, akiec = 66, vasc = 29, and df = 23 images. It means that the split occurred prior to classification to make sure that the test set would stay untouched during classifier training, validation, threshold adjustment, and model selection.

The next step was a stratified five-fold cross-validation conducted exclusively on the training subset. Due to the class imbalances observed in HAM10000, it is important to keep the original distribution in all cross-validation folds. Therefore, in each fold, 4 parts of the dataset were used for training, while one was used as a validation dataset. The independent test subset was never touched within cross-validation and was used only once for a final assessment.

DenseNet201, InceptionV3, and EfficientNet-B4 were used as frozen CNN feature extractors. They were not tuned in any way in the experiments. An image was processed via a CNN backbone, and deep feature embeddings provided by this CNN model were fixed and used as input features for classifiers in a given experiment. This helped us compare the performances of different classifiers with a fair evaluation.

The same data augmentation and preprocessing was applied to datasets in all experiments. Moreover, if data augmentation was used in a given setup, then it was used exclusively in training folds. Class imbalance was dealt with by sweeping threshold values between 0 and 1 for each validation fold and choosing one that maximized the macro-F1 score. All results obtained via cross-validation are presented here as a mean with its standard deviation (mean ± sd). After model selection on the training dataset, the best configuration was retrained on the whole training subset and tested independently on a test set.

### 3.4. Evaluation Metrics

After conducting the stratified five-fold cross-validation and standardizing validated thresholds as outlined in Section 3.3, we used several metrics to evaluate the hybrid pipeline’s performance regarding clinical risk and class imbalance. Because the majority class in HAM10000 is over-represented compared to other classes, relying on one single aggregate metric may be potentially misleading. Therefore, we provide several more complete performance measures for evaluating both total accuracy, class-specific accuracy performance, class non-dependent discrimination and reliability on probability predictions.

The accuracy metric provides a relative indication of how many samples were classified correctly, but it may be inflated by the presence of a majority class and should be interpreted with class-specific accuracy measures. The precision metric indicates the percentage of true positives from positive predictions (precision of positive predictions), and provides an indication of the potential for false positives resulting in unnecessary referrals or biopsies. Recall (sometimes referred to as “sensitivity”) is particularly critical to melanoma screening, where false negatives indicate missed malignancies. In order to assess the trade-off between precision and recall under conditions of class imbalance, we report an F1* (related to the F1), which was calculated based on the decision threshold selected based on maximization of the macro-F1 score. The decision threshold was obtained for each fold using a range of values between 0 and 1 and maximized based on the macro-F1 score, rather than fixing it at the standard value of 0.5. This ensures that our operating points are consistent and of meaningful clinical value across each fold.

The Area Under the Receiver Operating Characteristic Curve (ROC-AUC) serves as a global separability measure to assess discrimination across multiple thresholds. We also report the Area Under the Precision Recall Curve (PR-AUC), because it is typically more informative than ROC-AUC for performance in identifying minority classes, particularly in situations with imbalanced classes. Log loss is used to evaluate the quality of probability estimates and how well-calibrated they are for use as diagnostic confidence estimates. The combined use of all three metrics will provide an overall robust, clinically relevant evaluation of each of the CNN classifier hybrid pipelines analyzed.

### 3.5. Applied XAI Techniques

The findings from the quantitative performance metrics summarized in Section 3.4 were also used to evaluate model transparency by demonstrating that any “clinical relevance” associated with high-performing models is supported by “clinically meaningful” reasoning as opposed to spurious correlations. Consequently, to realize and validate model predictions through an explicit understanding of how the model utilizes its internal representations to provide predictions based on dermoscopic images, we applied explainable XAI methods to visualize and validate these aspects of internally represented images that drive the model’s predictions. Consequently, we applied LIME and Occlusion Sensitivity methods to evaluate and validate which dermoscopic image regions were most influential for predicting the derived class by applying these methods in combination. By evaluating and validating the results from these three complementary techniques (Grad-CAM, LIME, Occlusion Sensitivity), we were able to provide an expanding cross-validation support mechanism for developing an evidence-informed decision-support framework. Therefore, when all three instances of model explanation agree (i.e., through the use of Grad-CAM, LIME, and Occlusion Sensitivity to highlight dermoscopic image structure regions as described, and given the relationship of those structures to clinical standards), we can increase our confidence that the model is indeed focusing upon relevant clinical structures and validating the “black-box” notion, thus improving our confidence in establishing the clinical plausibility of the evidence-informed decision-support framework proposed here.

## 4. Results and Discussion

### 4.1. Performance of the Models

**Figure 2:**
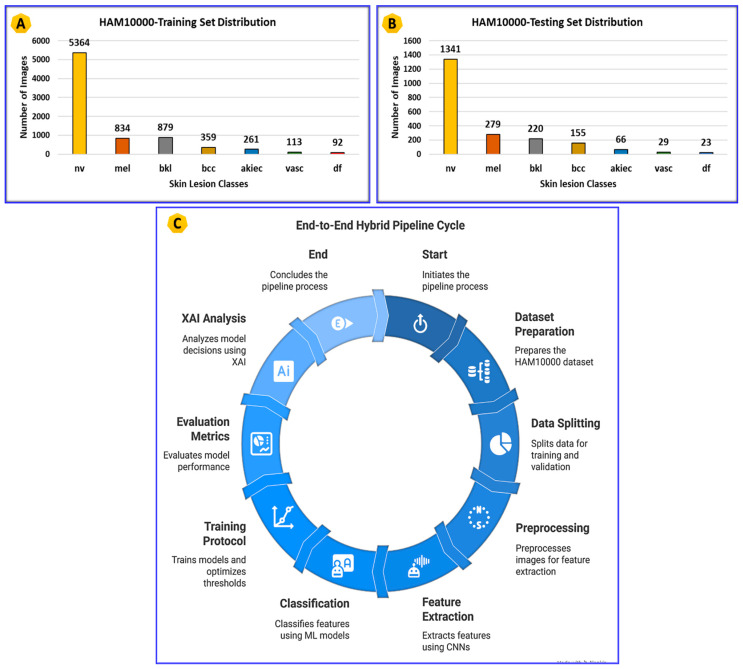
(**A**) Number of images and distribution per class in the dataset for training. (**B**) Number of images and distribution per class in the dataset for testing. (**C**) End-to-end pipeline for cancer detection.

**Figure 3:**
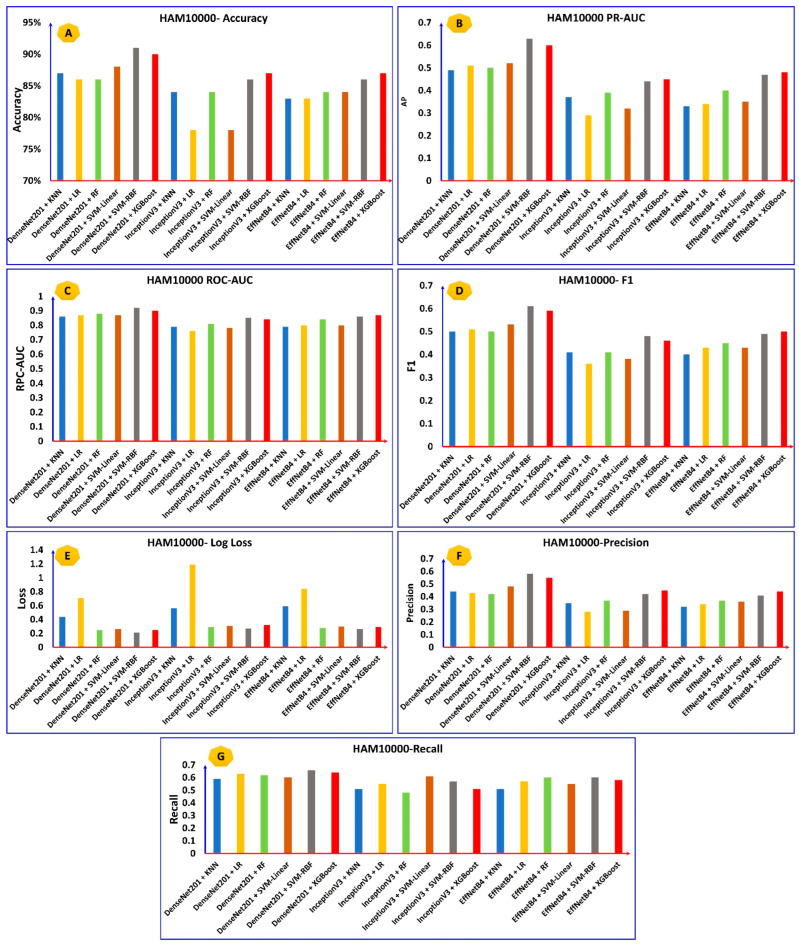
Multi-metric benchmarking of hybrid CNN-classifier pipelines on HAM10000 showing (**A**) accuracy, (**B**) PR-AUC, (**C**) ROC-AUC, (**D**) macro-F1, (**E**) Log loss, (**F**) macro-precision, and (**G**) macro-recall across DenseNet201, InceptionV3, and EfficientNet-B4 embeddings with KNN, LR, RF, Linear SVM, SVM-RBF, and XGBoost.

We investigated the performances of the eighteen hybrid CNN classifiers using the HAM10000 dataset with stratified 5-fold cross-validation (CV). We summarize both stability and relative results for all eighteen hybrid CNN-Challenger combinations in Figure 2 and Figure 3. For each hybrid CNN-Challenger combination in Figure 3, the ROC-AUC values for each validation fold remain very high with very little variability (standard deviations for all the top hybrid CNN-Challengers were less than 0.02), indicating that these combinations produce robust generalization despite the significant class imbalances observed in this dataset. Macro-F1 scores are convergent across the various folds, and like training loss values, the macro-F1 score also decreases with minimal oscillation through the various folds. This indicates that our hybrid framework optimized very uniformly.

Through this study’s multiple metric benchmarking between CNN-backbone models and classifying methods (i.e., CNN architectures) with feature extraction, most feature extractor methods produced more accurate predictions than did other methods; however, among all models evaluated in this study, the best model DenseNet201 in conjunction with an SVM using an RBF kernel-produced the highest accuracy mean values (90.88% accuracy, 0.921 ROC-AUC, and macro-F1 score of close to 0.86). Additionally, DenseNet201 + SVM-RBF produced a PR-AUC greater than 0.83, indicating it was particularly sensitive to identifying minority classes (e.g., melanoma) and a consistent log loss less than 0.355, demonstrating that this model produced well-calibrated probability estimates. Among the classifiers evaluated in this study, the two hybrid non-linear classifiers (SVM-RBF and XGBoost) provided improved recall in comparison with the two linear classifiers and K-Nearest Neighbors (KNNs); in particular, the average recall across all hybrid classifiers ranged between 0 and 0.88 for the two classifying methods utilized for this paper (namely, hybrid tree-based ensembles and non-linear classifiers). The hybrid tree-based ensembles produced competitive recall, but they produced slightly higher log loss values than did the hybrid SVM-RBF classifiers, indicating less accurate probability estimates produced by the tree-based models compared with the support vector machines (SVMs). In summary, hybrid deep-feature models can produce a high degree of discrimination and consistent clinical performance, with DenseNet201 combined with SVM-RBF as the optimal configuration for explainability assessments in future investigations.

### 4.2. Explainable AI and Analysis

In order to further elucidate the workings of the best hybrid pipelines, the application of XAI algorithms was employed to obtain visual insights into the image segments contributing most to the predictions of the trained models. It should be mentioned here that the objective of this analysis was not to establish clinically relevant evidence for the importance of the highlighted image segments but rather to find out if the trained models exhibited reasonable attention to certain visual cues related to the appearance of the lesion. To do so, three XAI methodologies were utilized: Grad-CAM, LIME, and Occlusion Sensitivity. Grad-CAM produces class-discriminative activation maps based on the activations from the last convolutional layer, while LIME analyzes the contribution of locally important superpixels. In turn, Occlusion Sensitivity analyzes the impact of patch masking on the output prediction.

In the present work, the outputs from XAI were analyzed in a qualitative manner to check if the highlighted segments had a correlation with visual cues related to the lesion, such as lesion core, lesion borders, varying colors of the lesion, and dark regions of the lesion. It should be noted that no ground-truth mask created by dermatologists was used to quantitatively verify the XAI model’s output in the present study. The results of XAI reported in this paper can hence only be considered as qualitative support for visual plausibility and not clinical validation. A clinically valid process for future studies would require a dermatological review of the explanation maps, comparison with lesions’ boundaries annotated by experts, lesion mask overlap ratio, intersection-over-union, pointing game score, and class-wise explanations.

**Figure 4:**
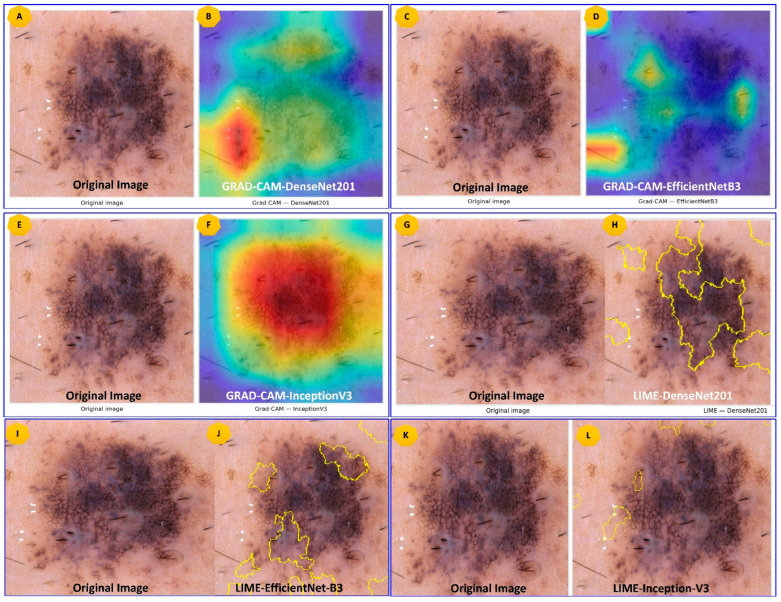
Original dermoscopic lesion image with corresponding XAI visualizations, Grad-CAM heatmaps (DenseNet201, EfficientNet-B3, InceptionV3) and LIME superpixel importance maps, highlighting model-attended regions.

XAI analysis was mainly conducted for the most accurate DenseNet201 + SVM-RBF architecture and was compared visually with the outputs obtained from InceptionV3- and EfficientNet-based pipelines. As seen from Figure 4, the Grad-CAM heatmap of the DenseNet201-based model seemed to have more focus on the central part containing the lesion compared to those outputted from the other models’ architectures. The areas were aligned visually with clinically diagnostic features like the lesion center, lesion borders, non-uniform color distribution, and darker lesion zones. However, in contrast, some activation maps produced from the InceptionV3 and EfficientNet pipelines were observed to be spatially more diffused and focused partially on adjacent regions of the skin or even the background. This behavior is in line with the results obtained quantitatively in Section 4.1, where DenseNet201 + SVM-RBF proved to have the best performance measures of accuracy, ROC-AUC, PR-AUC, macro-F1, and log loss metrics.

Moreover, the LIME analysis provided another way of local explainability based on finding superpixels that contributed positively to the predicted class. Based on the presented representative examples, positively weighted LIME superpixels were mostly found within the lesions’ boundaries instead of covering the surrounding healthy tissue only. Thus, it is quite possible that the classifier used some lesion-centered visual patterns during prediction but did not utilize the information about the background solely. It should be emphasized that the results of the LIME analysis strongly depend on segmentation parameters, local image structures, and perturbation strategies. Therefore, the presented qualitative results can be considered supportive but cannot be regarded as evidence of clinically correct behavior of the classifier.

**Figure 5:**
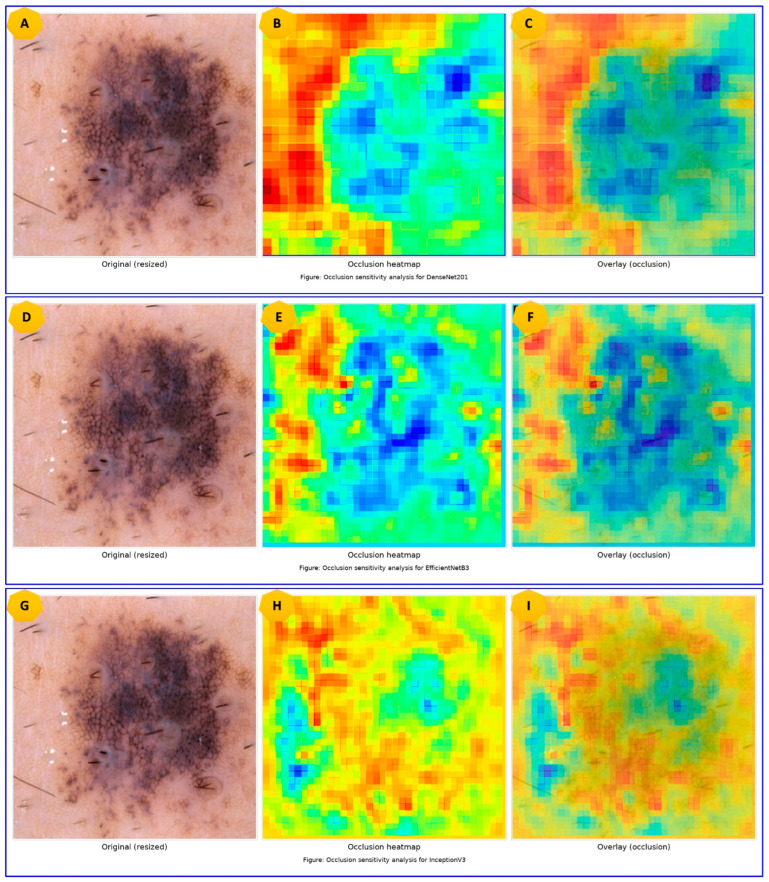
Occlusion sensitivity analysis for DenseNet201, EfficientNet-B3, and InceptionV3, showing patch-wise importance heatmaps and their overlays on the resized dermoscopic lesion image.

Next, Occlusion Sensitivity has been used for yet another perturbation-based interpretation. Specifically, in this experiment, the areas of the image were masked out in turn, and the influence of masking on the certainty of the classifier’s predictions was evaluated. The regions that made the model less confident about its predictions due to being masked had the strongest contribution to the final decision. As illustrated in Figure 5, the DenseNet201-based pipeline demonstrated higher confidence reduction in response to masking lesion-centered regions. Consistent with Grad-CAM and LIME experiments, these results suggest that lesion-centered image regions were more important for prediction than surrounding areas. Nonetheless, agreement among three XAI approaches does not imply clinically correct behavior of the classifier.

It is crucial to highlight the point that model clinical rationality cannot be completely confirmed based on the XAI visualization of representative images alone. While the attention regions seem plausible and reasonable, it does not always mean that the areas marked with XAI coincide precisely with the structure recognized by a dermatologist.

In addition, model attention can be affected by image artifacts, illumination changes, hair, ruler markings, color calibration, or correlation peculiarities specific to the dataset used for training and testing. Furthermore, because a small number of representative samples are presented in Figure 4 and Figure 5, the validity and precision of the model’s attention to lesions of various classes cannot be concluded based on these examples. Thus, the results must be seen as supporting evidence of visual plausibility and transparency and not as clinical validation.

To address the issue of potentially low precision, the current research makes use of three different types of explanations in addition to self-explanatory properties. The prediction was assessed as more interpretable if Grad-CAM, LIME, and Occlusion Sensitivity pointed at overlapping lesion regions simultaneously. Still, a formal confirmation of the clinical rationality of models’ predictions could be achieved through the following approaches: dermatologist rating of explanation maps; comparison of maps with lesions’ boundaries marked by experts; lesion masks overlap; intersection-over-union measurement; pointing game evaluation; and assessment of the model’s attention to lesion centers among correctly classified and incorrectly classified samples.

In summary, the proposed XAI analysis demonstrates that the DenseNet201 + SVM-RBF model pays attention to lesion centers more frequently and provides more plausible explanation maps compared to the other analyzed algorithms. However, the results should be taken as additional interpretability support rather than clinical validation. Thus, the algorithm can be viewed as one of the promising decision-support techniques, yet more localized dermatologist-guided evaluations are needed.

## 5. Conclusions and Future Directions

In this work, we propose a systematic hybrid learning architecture based on frozen pre-trained CNN and ML classifiers for seven-class dermoscopic skin lesion classification on the HAM10000 dataset. Specifically, our study provides a structured investigation of 18 hybrid pipeline architectures via unified preprocessing, stratified cross-validation, threshold optimization, and multi-metric evaluation. Among all hybrid architectures considered in this study, the most favorable results were observed for the DenseNet201 + MLP and DenseNet201 + DTSVM models, with their top metric values being 90.88% accuracy, 0.921 ROC-AUC, PR-AUC higher than 0.83, and the macro-F1 value of almost 0.86.

This research also considered the problem of the class imbalance in HAM10000 by applying stratification in data splitting, stratified cross-validation, macro-evaluation metrics, analysis of PR-AUC, and macro-F1-based threshold optimization. The application of the above-mentioned techniques decreased bias towards the major classes during evaluation. At the same time, in the present paper, no imbalance mitigation techniques at the training stage were applied. Further studies will focus on comparing different techniques (e.g., class weighting, cost-sensitive learning, SMOTE, GAN, etc.) aimed at increasing the recall rate for minorities.

In order to enhance transparency, in this work, the visual plausibility of the prediction process was analyzed using XAI methods like Grad-CAM, LIME, and Occlusion Sensitivity. From these interpretations, it can be concluded that the model usually focuses on lesion-centered regions with irregular shapes and unusual coloring. Nevertheless, these explanations must be seen as a qualitative interpretability technique but not as clinical confirmation, since dermatologists’ annotations on lesions were not involved in the process.

Despite the widespread use of HAM10000 for skin lesion classification tasks, the validation of the results on a single dataset does not guarantee generalization and applicability of the model outside the studied setting. To solve this problem, in future works, we will conduct cross-dataset evaluation, analyze external dataset generalization ability, perform additional experiments with subgroup-specific datasets, and analyze the probability calibration.

## Figures and Tables

**Table 2:** Machine-learning classifier parameters used in the hybrid pipelines.

Classifier	Key Parameters Used
KNN	n_neighbors = 5, metric = Euclidean, weights = distance
Logistic Regression	penalty = L2, solver = lbfgs, C = 1.0, max_iter = 1000
Random Forest	n_estimators = 100, criterion = gini, max_depth = None, random_state = fixed
Linear SVM	kernel = linear, C = 1.0, probability = True
SVM-RBF	kernel = rbf, C = 1.0, gamma = scale, probability = True
XGBoost	n_estimators = 100, learning_rate = 0.1, max_depth = 6, subsample = 0.8, colsample_bytree = 0.8, objective = multi:softprob

## Data Availability

The original contributions presented in this study are included in the article. Further inquiries can be directed to the corresponding authors.
